# Preparation and Characterization of Cu and Ni on Alumina Supports and Their Use in the Synthesis of Low-Temperature Metal-Phthalocyanine Using a Parallel-Plate Reactor

**DOI:** 10.3390/ma6104324

**Published:** 2013-09-30

**Authors:** Fernando Sánchez-De la Torre, Javier Rivera De la Rosa, Boris I. Kharisov, Carlos J. Lucio-Ortiz

**Affiliations:** 1 Universidad Autónoma de Nuevo León, UANL, Facultad de Ciencias Químicas, Ave. Universidad S/N, Cd. Universitaria, San Nicolás de los Garza, N. L. 64451, Mexico; E-Mails: ferska67@hotmail.com (F.S.-D.T.); bkhariss@hotmail.com (B.I.K.); luciomex@hotmail.com (C.J.L.-O.); 2 Universidad Autónoma de Nuevo León, UANL, Centro de Innovación, Investigación y Desarrollo en Ingeniería y Tecnología (CIIDIT), Km 10 de la nueva carretera al Aeropuerto Internacional de Monterrey, PIIT Monterrey, Apodaca, Nuevo León 66600, Mexico

**Keywords:** Ni/Al_2_O_3_, Cu/Al_2_O_3_, phthalocyanine, phthalonitrile, Ni-phthalocyanine, Cu-phthalocyanine, parallel-plate reactor

## Abstract

Ni- and Cu/alumina powders were prepared and characterized by X-ray diffraction (XRD), scanning electronic microscope (SEM), and N_2_ physisorption isotherms were also determined. The Ni/Al_2_O_3_ sample reveled agglomerated (1 μm) of nanoparticles of Ni (30–80 nm) however, NiO particles were also identified, probably for the low temperature during the H_2_ reduction treatment (350 °C), the Cu/Al_2_O_3_ sample presented agglomerates (1–1.5 μm) of nanoparticles (70–150 nm), but only of pure copper. Both surface morphologies were different, but resulted in mesoporous material, with a higher specificity for the Ni sample. The surfaces were used in a new proposal for producing copper and nickel phthalocyanines using a parallel-plate reactor. Phthalonitrile was used and metallic particles were deposited on alumina in ethanol solution with CH_3_ONa at low temperatures; ≤60 °C. The mass-transfer was evaluated in reaction testing with a recent three-resistance model. The kinetics were studied with a Langmuir-Hinshelwood model. The activation energy and Thiele modulus revealed a slow surface reaction. The nickel sample was the most active, influenced by the NiO morphology and phthalonitrile adsorption.

## 1.Introduction

The design of successful pigments demands a deep knowledge of new synthesis routes. Different synthetic routes for producing metal-free phthalocyanine (H_2_Pc) or metal phthalocyaninates (MPc) at low temperatures (20–50 °C), from phthalonitrile, have been reported, including a direct electrochemical procedure that uses sacrificial metallic anodes or metal salts [1,2]; the use of solid strong bases [3]; UV irradiation of the reaction system [4,5]; and the use of elemental metals [6,7,8]. At low temperatures, an additional “impulse” is needed for the cyclization process, the surface energy of a strong base (solid CH_3_ONa) [3], the extra energy of defects at the surface of elemental metals [7] or zeoles [9] could serve as an impulse to reach the energy barrier. Thus, any source of additional energy could improve the success of the synthesis at low temperatures. Phthalonitrile is an expensive precursor for the phthalocyanine industry, compared to urea and phthalic aydride, but it is important to the exploration of these different processing routes according to the principles of Green Chemistry [10], which include the use of green solvents, such as water or ethanol. However, obtaining phthalocyanines from the cheapest precursors requires relatively extreme conditions (>180 °C, use of catalysts and promoters). In contrast, the use of phthalonitrile allows quantitative-yield reactions at milder conditions (<130 °C). Further decreasing the temperatures to ambient conditions would yield energy savings when using this precursor to produce phthalocyanine, which would offset its high cost.

In the typical synthesis of copper phthalocyanine (CuPc), phthalic anhydride, urea, and copper (I) chloride are heated in a batch reactor system at a ratio of 1:16:16, respectively, in a high-boiling-point solvent, such as trichlorobenzene, nitrobenzene, or kerosene. The solvent is removed after the formation of copper phthalocyanine [11].

Depositing copper and nickel particles on alumina supports on parallel plates to react with the fluid-phase phthalonitrile in the reactor parallel plates has the advantage of eliminating the need to separate the solid products (NiPc or CuPc) from the metal-alumina solid.

Our research group has, thus far, reported the synthesis and characterization of Cu and Ni supported on alumina and their reaction at only one temperature, in flasks, to produce NiPc and CuPc at low temperatures [12]. It is important to replicate the synthesis of metallic particles, since this time the morphological changes can influence on the new arrangement of the reactor, which is continuous flow, as the particles are supported on the parallel plates. Due to the promising results obtained in that study, the process should be scaled up to a flow reactor before improving the material. Catalyst systems are encountered in a wide variety of designs, and catalysts (e.g., pelletized catalysts) can serve as their own support material. However, the mass-transfer resistance is significant in large particles, and a significantly larger pressure drop is experienced in a fixed bed of smaller particles. In the reactor presented in this work, the effect of mass transfer in a parallel-plate system, which may reduce the surface reaction, must be evaluated.

### 1.1. Three-Resistance Mass-Transfer Model

Recently, Joshi *et al.* [13,14] reported the use of internal mass-transfer coefficients in the modelling of the diffusion and reaction in catalytic monoliths; a three-resistance model is more useful and important than the classical effectiveness-factor concept. They considered various washcoat and channel geometries, including parallel plates.

**Figure 1:**
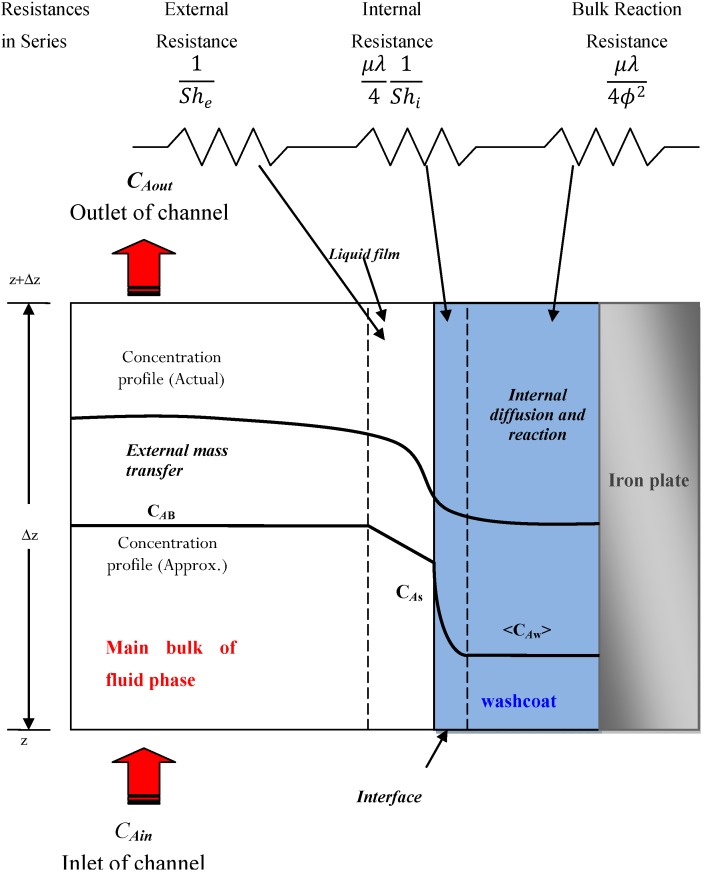
Schematic diagram of the control volume that illustrates the fluid-solid mass transfer during a chemical reaction in the washcoat with resistances using a series approach.

Figure 1 (adapted from reference 13) presents a control volume that represents the concentration profile of substance *A*; which is a reagent that enters the channel with an inlet concentration, *C_Ain_*; and exits with a lower concentration, *C_Aout_*, by reaction conversion. As evident in the figure, the concentration on the surface, *C_As_*, is the first real contact between substance *A* and the catalyst material that can find an active site; substance *A* can then be introduced by diffusion into the material interface and is later considered to internally diffuse and react in the bulk catalyst at a constant concentration of *C_Aw_*. The three resistances are defined as follows. The first resistance is an external resistance that is driven by the difference in the surface concentration (*C_As_*) and the bulk concentration of species *A* in the fluid phase (*C_AB_*), which is the average of *C_Ain_* and *C_Aout_*. The second resistance occurs at the interface, which is defined by the surface at some depth within the catalytic material that contains a concentration profile between *C_As_* and the concentration in the bulk catalyst (*C_Aw_*). The third resistance is the reaction resistance of diffusion into the pores of the washcoat catalytic material anchored to the wall channel (an iron plate in the parallel-plate reactor).

The Thiele modulus (ϕ), which is the ratio of the intra-particle diffusion time to the characteristic reaction time, was used to study the changes in the resistances. The generalized Thiele modulus was calculated in this work, which Petersen [15] defined for a single-step irreversible reaction as follows:

(1) $$\phi_{1}^{2}=\frac{2R_{{\text{Ω}}_{2}}^{2}\left[\int_{0}^{C_{s}}R\left(C´\right)dC\right]}{D_{e}C_{s}^{2}}$$

### 1.2. Effective Diffusivity

ements of the effective diffusivity in the catalytic washcoat, via measurements of the pore size distribution and the respective pore volume in the structure, were necessary to utilize the models. The effective diffusivity is usually related to the diffusion coefficient in the pores via the porosity of the solid, ε, and the tortuosity of the pores, τ. The parallel-pore model represents a general relationship between the effective diffusivity and the pore diffusivity. Zhang *et al.* [16] demonstrated that the one- and two-dimensional mathematical models used to calculate the effective diffusivity in the catalyst/washcoat layer are applicable to a porous monolith support, e.g., cordierite.

(2) $$D_{e}=\frac{\varepsilon }{\tau }D_{p}$$

The diffusion coefficient in the pores, *D_p_*: can be determined from the Knudsen diffusion coefficient, *D_K_*, and the bulk diffusion coefficient, *D_AB_*:

(3) $$\frac{1}{D_{p}}=\frac{1}{D_{K}}+\frac{1}{D_{AB}}$$

*D_K_* depends on the temperature, the molar mass of the diffusing species and the pore diameter, *d_p_*. Therefore, a narrow pore-size distribution is important:

(4) $$D_{K}=48.5\;d_{p}\sqrt{\frac{T}{M}}$$

The value of *D_AB_* in a liquid system can usually be calculated by

(5) $$D_{AB}=\frac{\left(117.3\times 10^{-18}\right){\left(\varphi M_{B}\right)}^{0.5}T}{\mu_{sol}\upsilon_{A}^{0.6}}$$

In their report on catalyst-coated monoliths, Kolaczkowski *et al.* [17] noted that a parallel model provided tortuosity factors of 8.5 and 8.1, and used a chromatographic method to evaluate the effective diffusivity in a monolith. They used a tortuosity factor of 6 and a washcoat porosity (ε) of 0.67. In this work, *D_e_* was evaluated using Equations (2)–(5) for the Ni and Cu particles on alumina powder (catalyst washcoat) coated on the iron plates of a parallel-plate reactor. The Ni/Al_2_O_3_ and Cu/Al_2_O_3_ washcoats were assumed to have pore diameters of 8.8 and 6.0 nm, respectively. However, the pore size of the washcoat material, as evaluated by scanning electronic microscopy (SEM), must be considered; the values of μ = *D_AB_*/*D_B_* ranged from 12.749 to 12.752 for temperatures ranging from 30 to 50 °C when ε = 0.67 and τ = 8.5. The μ relationship affects the reaction and internal mass-transfer resistances.

### 1.3. Mass-Transfer Resistances

As evident from Figure 1 and the different Sherwood (*Sh*) numbers in Equation 6, the mass transfer is considered to be a series of resistances:

(6) $$\frac{1}{Sh_{app}}=\frac{1}{Sh_{e}}+\frac{\mu \lambda }{4}\frac{1}{Sh_{i}}+\frac{\mu \lambda }{4\phi^{2}}$$

The following assumptions were made: (i) the flow is laminar and fully developed; (ii) convection dominates axial diffusion (*i.e.*, the axial Peclet number is very large); and (iii) the reactant inlet concentration is low enough that the adiabatic temperature increase is small, which allows the channel to be considered isothermal and the temperature dependence of the physical properties to be neglected. The Sherwood numbers (in external and internal resistances) must be estimated using correlations. The correlation for *Sh*, given by West *et al.* [18], is a simple approximation for *Sh* that is valid for any arbitrary geometry that can be obtained by a simple combination of the small and large transverse Peclet number asymptotes. This correlation can be used to estimate the external Sherwood number (*Sh_e_*) from Equation 7.

(7) $$Sh\left(L\right)=\{\begin{array}{c} 1.4P^{1/2}Sc^{-1/6}=\;\;0.35{\left(\frac{D_{h}}{L}\right)}^{1/2}Re^{1/2}Sc^{1/3}\\ Sh_{\infty } \end{array}for\;\begin{array}{c} P>0.51Sh_{e\infty }^{2}Sc^{1/3}\\ P<0.51Sh_{e\infty }^{2}Sc^{1/3} \end{array}$$

Joshi *et al.* [13] used an expression that related the Sherwood number to the internal resistance (*Shi*) and the Thiele modulus (ϕ), as shown in Equation 8:

(8) $$Sh_{i}=Sh_{i\infty }+\frac{\Lambda \phi^{2}}{1+\Lambda \phi }$$

The constant *Λ* depends of the washcoating geometry and the kinetic parameters. Joshi *et al.* [13,14] presented a table that lists the numerically computed values of *Sh*_*i*∞_ and the constant *Λ* for various washcoat-channel geometries for the case of first-order kinetics. Notably, *Sh_i_* = *Sh*_*i*∞_ for a slow reaction (ϕ << 1) in Equation 7. Based on Equations 6–8, *Sh_i_* can be combined with the traditional external mass-transfer coefficient (*Sh_e_*) to obtain the overall mass-transfer coefficient (*Sh_app_*), which is an experimentally measurable quantity that depends on the catalyst activity profile, the washcoat and channel geometries, and the species diffusivities in the fluid phase and washcoat.

The third term on the right side of Equation 6 refers to the bulk reaction resistance, which depends on the internal diffusion, the reaction and the square of the Thiele modulus. The square of the Thiele modulus is the ratio of two characteristic times: the diffusion and reaction times. When the Thiele modulus is large, internal diffusion usually limits the overall rate of reaction; when ϕ_1_^2^ is small, the surface reaction is usually rate-limiting. In this work, the Thiele modulus was calculated using Equation 1.

This study used the phthalonitrile conversion experimental data of the batch reactor for both materials, Ni/ and Cu/Al_2_O_3_, at 30, 40, and 50 °C to determinate a simple kinetic model and its parameters. The kinetic model was used to calculate the Thiele modulus in the washcoat channel of the parallel-plate reactor experiments.

This work aimed to correlate the characterisation of Ni and Cu nanoparticles supported on alumina with their use in a new parallel-plate reactor arrangement to produce Ni and Cu phthalocyanines at low reaction temperatures (≤60 °C) using phthalonitrile in an ethanol solution. Furthermore, this study evaluated the mass transfer using the new three-resistance model. Finally, the kinetic data from parallel-plate reactor tests were studied using a Langmuir-Hinshelwood mechanism to better understand the surface reaction by analyzing the kinetic parameters.

## 2. Results and Discussion

### 2.1. XRD Patterns

**Figure 2:**
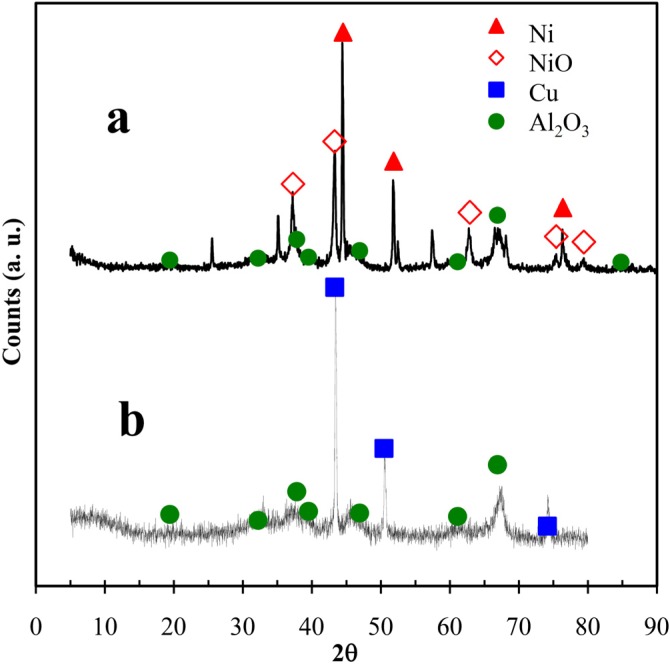
XRD patterns of prepared powders of (**a**) Ni; and (**b**) Cu particles on alumina.

Figure 2 presents the XRD patterns of both copper and nickel supported on alumina powders. The nickel on alumina sample (Figure 2a) contained crystalline elemental nickel (JCPDS 4-0850) and nickel oxide (JCPDS 02-7440). A significant amount of NiO was not reduced to elemental Ni because the treatment temperature during the sample preparation was insufficient for total conversion. Considering the relative maximum diffraction peaks for Ni (2θ = 44.505°) and NiO (2θ = 43.276°), these crystalline phases comprised 66% and 34% of the total sample, respectively. Previous studies have reported temperatures below 450 °C for impregnated nickel oxide (obtained similarly to that in this work) on alumina, which indicates a weak interaction with alumina [19]. The reduction temperature for our sample (Ni/Al_2_O_3_) was 350 °C, suggesting that many Ni and NiO particles were available on the surface. NiO nanoparticles deposited on mesoporous surfaces have been used in liquid-phase catalytic reactions for compounds such as ortho-aminophenol and have exhibited a high affinity to adsorption due to the adjacency of the amino and hydroxyl groups [20]. This work used phthalonitrile, which contains two adjacent nitrile groups. The formation of nitrile groups is favored on NiO particles [21] and can result in an adsorption-desorption equilibrium.

The sample of copper on alumina (Figure 2b) contained an elemental copper crystalline phase (JCPDS 04-0836) but no oxide copper crystalline species. The maximum diffraction peak (2θ = 43.30°) of the Cu crystalline phase presented a high number of counts, indicating a high crystallinity. The γ-Al_2_O_3_ crystalline phase (JCPDS 01-074-2206) was detected for the alumina support of both samples. However, this crystalline phase of alumina is known to have catalytic properties due to its mesoporosity and acid active sites, such as Brönsted and Lewis acids, which are necessary in certain reactions to achieve skeletal rearrangements [22], although they may be somewhat inhibited by depositing Ni and Cu particles. A mesoporous structure promotes percolation by increasing the permeability; as such, a significant amount of reactant can be diffused well into the particle interior to reach the active sites [9,23], e.g., phthalonitrile reaching the nickel or copper particles in this study.

### 2.2. SEM Images

**Figure 3:**
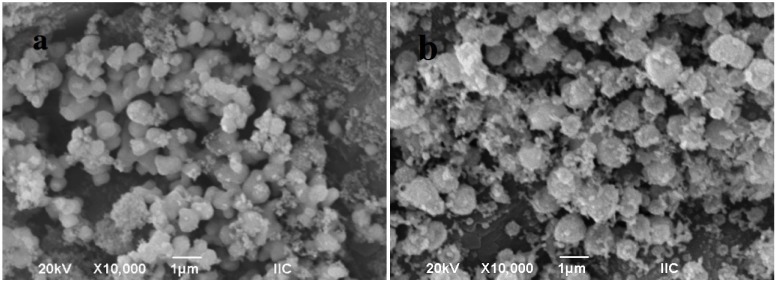
SEM micrographs of (**a**) Ni; and (**b**) Cu particles on alumina.

Figure 3 presents the scanning electron photomicrographs of copper and nickel on alumina substrates. The SEM images of the Ni/Al_2_O_3_ sample (Figure 3a) reveal two types of nickel particles on the alumina surface. One type consisted of nearly spherical grains with slightly deformed and softened contours. Some of these particles were connected by incipient melting. These particles ranged from 150 to 600 nm in size and corresponded to NiO species [24]. The other type of nickel particles were much smaller in size (approximately 30 to 80 nm) and showed a poorly defined morphology. This type corresponded to elemental Ni [25], and these nanoparticles were incorporated into upper alumina and NiO grains to form agglomerates of up to 1 μm in diameter.

The micrograph of the Cu/Al_2_O_3_ sample (Figure 3b) shows the alumina grains with the copper particles, which displayed the same morphology as the nickel sample. The nanometric (~70–150 nm) elemental copper particles were present as individual particles and as agglomerations (1–1.5 μm) on the alumina support. Their dispersion was better than that of the Ni/Al_2_O_3_ sample. The mean size of crystallite was determined from the line broadening of the XRD peak of Cu (111) (2θ = 43.30 in Figure 2) using the Scherrer Equation [26], which yielded a value of 169 nm. This finding supports the general observation that individual metallic copper particles dispersed on supports usually have crystallite sizes similar to the mean particle size [27,28].

### 2.3. Textural Analysis from N_2_ Isotherms

**Figure 4:**
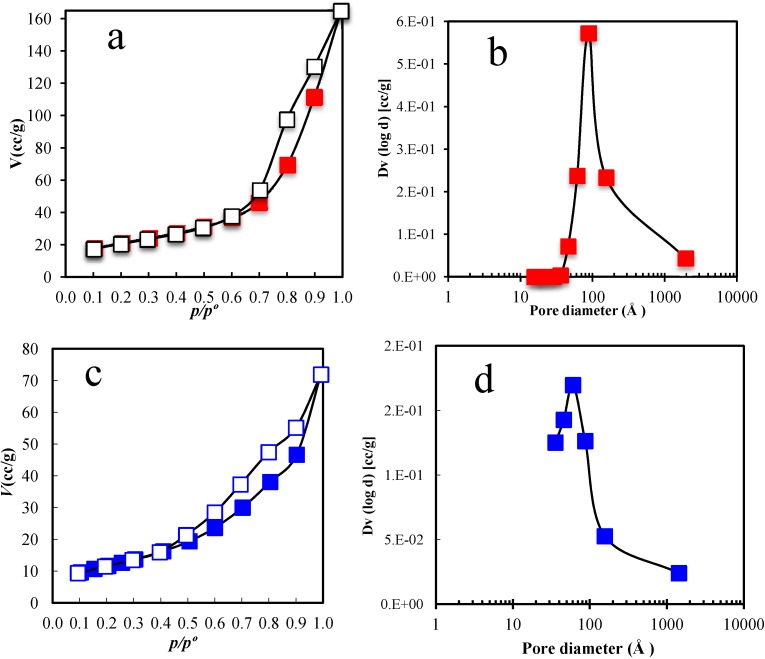
Adsorption-desorption isotherms of (**a**) Ni/Al_2_O_3_; (**c**) Cu/Al_2_O_3_; and the PSD of (**b**) Ni/Al_2_O_3_; and (**d**) Cu/Al_2_O_3_.

Figure 4 presents the N_2_ adsorption-desorption isotherms at 77 K and the pore size distribution (PSD) according the Barrett-Joyner-Halenda (BJH) method for the Ni/Al_2_O_3_ and Cu/Al_2_O_3_ samples. In the adsorption-desorption isotherms, filled and open symbols indicate the adsorption and desorption values, respectively. The International Union of Pure and Applied Chemistry (IUPAC) classifies the shapes of the adsorption isotherms for both samples (Figure 4a,c) as type IV [29] with a hysteresis loop, which is associated with capillary condensation in the mesopores. The initial part of the isotherm (until *p*/*p*° 0.5) can be attributed to monolayer-multilayer adsorption because it follows the same path of desorption, which demonstrates weak adsorbate-adsorbent interactions. The hysteresis loop is of type H3, which is usually indicative of aggregates of platelet particles or adsorbents containing slit pores. The hysteresis loop does not exhibit limiting adsorption at high *p*/*p*° values, which agrees with the SEM images. This phenomenon is related to the presence of agglomerates, *i.e.*, an assembly of particles rigidly joined together. Figure 4b shows the PSD Ni/Al_2_O_3_ sample, which exhibited a homogenous monomodal distribution. Although the NiO and Ni particles were deposited on the alumina substrate, which could have blocked the alumina pores, the PSD of the final material was uniform. This uniformity is very important to the phthalonitrile reactant in ethanol solution, which can then easily and homogeneously contact the elemental nickel and include it in the cyclization reaction.

Figure 4c shows the adsorption-desorption isotherm for the Cu/Al_2_O_3_ sample. Although the same weight percentages of copper and nickel were deposited on the alumina. Figure 4c clearly shows that less nitrogen was adsorbed on Cu/Al_2_O_3_ than on Ni/Al_2_O_3_ (60 and 170 cc/g, respectively). This finding can be attributed to the good surface coverage of alumina and the pore blocking due to the nanometric size and homogeneous morphology of some pure copper particles observed in the SEM images and XRD pattern. The IUPAC classifies the adsorption curve as type IV and the hysteresis loop type as H3, which is similar to the Ni/Al_2_O_3_ case. The PSD shows (Figure 4d) the absence of pores smaller than 35 Å. The most prevalent pore size was 65 Å, few pores were greater than 200 Å. γ-Alumina typically presents a mixture of mesopores and macropores [30]; apparently, the copper particles blocked the macropores and some larger mesopores (500 to 200 Å), leaving the smaller mesopores sufficiently accessible for the percolation of reactants.

Table 1 reports the microstructural porosity properties of both samples, and shows the mean size of crystallite calculated with the Scherrer Equation for both metals deposited on alumina. The specific area calculated by the Brunauer, Emmett, and Teller (BET) method for both samples proves that the mixture of NiO and Ni particles in the Ni/Al_2_O_3_ sample provided a greater available area than the nanosized copper particles in Cu/Al_2_O_3_. The reported pore size corresponds to the PSD mode (Figure 4b,d). The interaction of the phthalonitrile solution in alcohol with the nickel sample was expected to be better than that with the copper sample; however, the reaction rate depends on more than just the adsorption and permeability (amount of fluid penetrating through a structure). In this case, the metal particles also acted as a reactant to produce the MPc. As the crystallite size of nickel was smaller than that of copper according the XRD patterns, the use of nickel may be advantageous.

When metallic particle is used in the reaction, the reaction must be initiated by a nucleophilic attack on one of the phthalonitrile moieties, after which the Cu or Ni atom oxidizes to an ion species and contributes two electrons to the bond formation [31]. As this nucleophile is regenerated at the end of the reaction, it is only needed in catalytic amounts. In this work, the nucleophile was CH_3_ONa.

**Table 1:** Characteristics of nickel and copper particles deposited on alumina samples.

Sample	Mean size of crystallite of pure metal (nm)	A_BET_ (m^2^/g)	BJH desorption pore size, mode (Å)
Ni/Al_2_O_3_	46.92	88.06	88.06
Cu/Al_2_O_3_	169.26	43.01	60.57

### 2.4. Kinetic Model in the Reaction Batch Reactor

The experimental data for the reaction performed in a batch reactor were used to determine a kinetic expression for the reaction. The conversion data for a synthesis of acid phthalocyanine (H_2_Pc), which used phthalonitrile/ethanol on natural zeolites, were analyzed in a previous study to deduce a second-order reaction [9]. In this work, the first- and second-order reaction rate expressions were fitted to the reaction data of batch experiments. The best fit was achieved for second-order reaction kinetics. Equation 9 shows the second-order expression.

(9) 1CA0x1−x=ρBA0exp(−EaRT)t

Table 2 compiles the activation energies of both materials studied in the second-order kinetic expression. The activation energies for both metals were low, indicating that a transport mechanism was more prevalent than an entirely reactive mechanism [32]. However, low activation energies have also been reported for cyclization reactions [33] and the low conversion of acid phthalocyanine formation after 24 h [9], which agrees with the experimental conversion data obtained in this work (Table 2). Interestingly, both activation energies were very similar, but the pre-exponential value for Ni/Al_2_O_3_ was four-fold higher than that for Cu/Al_2_O_3_. The Ni particles yielded a higher conversion than the Cu particles (Table 2). This improved reactivity can be attributed to the standard electrode potential of Ni^2+^, which is lower than for Cu^2+^ ($E_{Ni^{2+}/Ni^{0}}^{0}$ = 0.25V$E_{Cu^{2+}/Cu^{0}}^{0}$ = 0.34 V). Thus, elemental nickel could be easier to oxidize than elemental copper for an oxidizing agent in an ethanolic medium depending on the concentrations.

The cyclization mechanism can be described as follows: the nucleophile (methoxide anion) attacks a nitrile group that acts as an electrophile at the carbon atom to form a bond with this carbon. This bond moves one of the triple bonds of the nitrile group to the carbon of the second nitrile group of the same phthalonitrile molecule. Later, one of the triple bonds of this second nitrile group moves to the nitrile group of a second phthalonitrile molecule and, thus, continues to propagate and, thus, form four pyrroles that bond the four phthalonitrile molecules. To regenerate the nucleophile (leaving the cyclic molecule), the metal must be oxidized by either nickel or copper to shear two electrons with two negatively charged nitrogen atoms of two pyrrole groups and form two additional conjugated bonds with the two nitrogen atoms of the other two pyrrole groups. Aravindakshan presented a mechanistic scheme figure [31].

**Table 2:** Kinetics and mass transfer parameters.

Batch reactor				Parallel-plate reactor						
**Conditions**	24 h phthalonitrile conversion %. At *T* = 30, 40, 50 °C	*ρ_B_Ao* L/(mol-s)*	*Ea* J/mol	Thiele modulus (ϕ)^**^			*k*^0^ mol*_A_*/g*_cat_*-s	*Ea_P-PR_* J/mol	$∆H_{ads}^{0}$ J/mol	$∆S_{ads}^{0}$ J/mol-K
				*T* = 30 °C	*T* = 40 °C	*T* = 50 °C				
**Ni/Al_2_O_3_**	4.7, 6.74,8.39	0.1658	24,615	1.015 × 10^−2^	1.403 × 10^−2^	1.800 × 10^−2^	3.187 × 10^−2^	21,475	−10,374	−59.47
**Cu/Al_2_O_3_**	2.19, 3.00, 3.79	3.784 × 10^−2^	22,939	4.523 × 10^−3^	6.046 × 10^−3^	7.695 × 10^−3^	3.300 × 10^−3^	13,604	−7,190	−60.54

* Taking *ρ_B_* = 0.5 g*_cat_*/0.125 L; ** Calculate at conversion archived at 24 h.

### 2.5. Evaluation of the Mass-Transfer Effect

**Figure 5:**
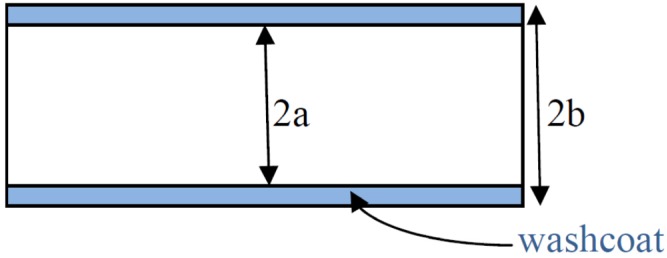
Dimensions of a channel of parallel-plate reactor with a washcoat applied to calculate the λ parameter. $R_{\Omega_{1}}$ = a; $R_{\Omega_{2}}$ = *b* – *a*; and $\lambda =\frac{R_{\Omega_{2}}}{R_{\Omega_{1}}}$.

**Figure 6:**
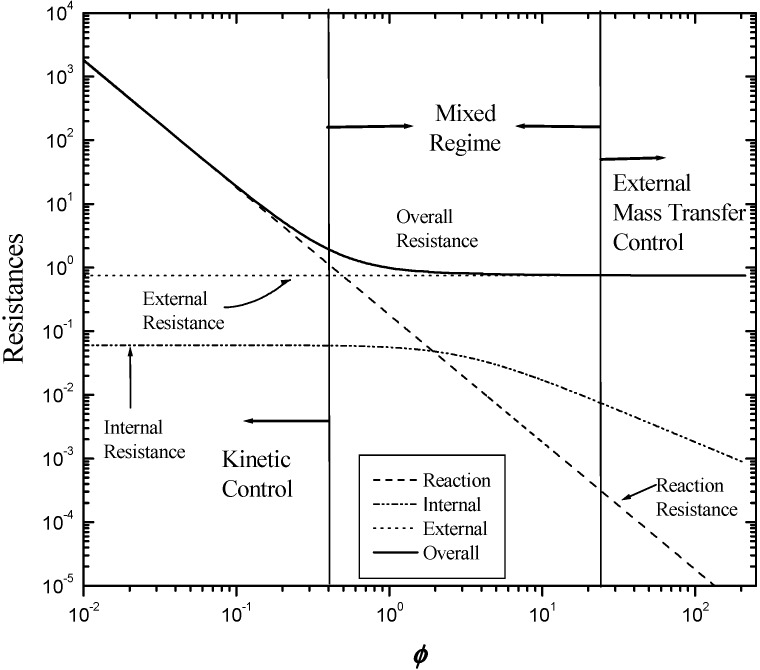
Overall resistance and its three individual resistances as function of the Thiele modulus and the three controlling regimens. For μ = *D_f_*/*D_e_* = 13.05 and λ = ${\text{R}}_{\Omega_{2}}$/${\text{R}}_{\Omega_{1}}$ = 0.05.

Figure 5 presents a diagram of a parallel-plate channel and the dimensions, taking into account the calculated parameter λ. Figure 6 presents the various resistances included in Equation 6, which considers the mass-transfer phenomena involved in the chemical reaction of the deposition of a washcoat into a channel as a function of the Thiele modulus. In this case, the data for the Ni/Al_2_O_3_ washcoat at 30°C were analyzed. The values of λ and μ used were 0.0556 and 12.750, respectively, which indicates that the length scale for the transverse diffusion for the washcoat was 5% of that for the transverse fluid phase and that the bulk diffusion coefficient was 12 times greater than the effective diffusivity (which includes the porosity and tortuosity) over the total cross-sectional area of the porous layer. The external Sherwood number (*Sh_e_*) was constant because the Sherwood number depends on the constant values of *Re* and *Sc* and not the Thiele modulus at constant temperatures if the transversal Peclet number (*P*) is greater than $0.51Sh_{e\infty }^{2}Sc^{1/3}$ , as is the case here (Equation 7) [18]. Equation 8 was used to evaluate the internal Sherwood numbers using *Sh_i_*_∞_ and *Λ* values of 3 and 0.32, respectively. The criteria for the kinetic and mass-transfer control boundaries were the same as those used by Joshi *et al.* [13].

The Thiele modulus was calculated using Equation 1, and the reaction rates used to compute the Thiele modulus were obtained using the second-order reaction ratmEquation 9).The kinetic parameters of this model are reported in Table 2. The values of the Thiele modulus at the three temperatures for both washcoat systems (Ni/ and Cu/Al_2_O_3_) in a parallel-plate reactor are reported in Table 2. The values of the Thiele modulus for both washcoat materials were less than 10^−1^, which is representative of slow reactions. As shown in Figure 6, the reaction was kinetically controlled. The kinetics can be evaluated using the experimental data from the parallel-plate reactor tests under the conditions mentioned above.

### 2.6. Kinetic Model in a Parallel-Plate Reactor

**Figure 7:**
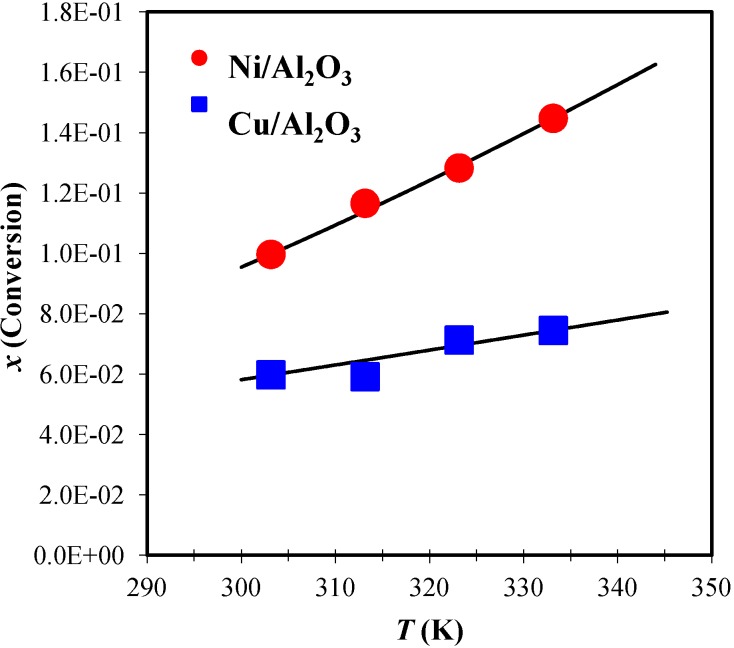
Conversion fraction at 24 h as a function of the temperature for a parallel-plate reactor for both metals supported on alumina powder adhered to the plates.

Figure 7 presents the experimental conversion fraction data as a function of the temperature of reaction in a parallel-plate reactor. Taking the phthalonitrile as limiting reagent the percentage of yield at 30, 40, 50, and 60 °C for Cu-phthalocyanine were 5.97%, 5.91%, 7.13%, and 7.45%, and for Ni-phthalocyanine were 9.97%, 11.66%, 12.86%, 14.47%, at 24 hours of reaction. The yields are low, as phthalonitrile initial concentrations were low, but it was important to maintain the same proportions of precursors used in the batch reactor, as the batch kinetic data were used to calculate the Thiele modulus (Equation 1). In other hand, low conversions are very convenient to study the kinetic, since to calculate the surface reaction rate value ($-{r´}_{A}$) it is necessary to use plug-flow design equation as $-{r´}_{A}≅F_{Ao}\left(x_{A}\right)/W_{cat}$. The experimental conversion data were fitted to a Langmuir-Hinshelwood model via Equation 10 (continue lines in Figure 7).

(10) $$-{r´}_{A}=\frac{kK_{A}C_{A}}{\left(1+K_{A}\right)}$$

The model considers the reversible adsorption of phthalonitrile and the reaction rate on the surface, which is the controlling step. Table 2 presents the reaction rate constant (*k*) according to the Arrhenius Equation:

(11) $$\text{ln}k=\text{ln}k^{0}-\frac{Ea_{P-PR}}{RT}$$

The adsorption constant (*K_A_*) was analyzed according to the van’t Hoff Equation:

(12) $$\text{ln}K_{A}=-\frac{∆H_{ads}^{0}}{RT}+\frac{∆S_{ads}^{0}}{R}$$

The pre-exponential factor, activation energy, standard enthalpy, and entropy properties were used to assess the fit of the constant parameters with the experimental data. Table 2 shows the obtained values. The pre-exponential factor for the Ni/Al_2_O_3_ reaction was greater than that obtained for Cu/Al_2_O_3_, and its obvious consequence is the higher conversions observed in Figure 7 for Ni/Al_2_O_3_. For both metals, the activation energy was lower than that obtained for the batch reactor. However, the values were very similar for Ni/Al_2_O_3_, whereas the activation energy for Cu/Al_2_O_3_ considerably decreased. The surface reaction on Ni/Al_2_O_3_ appeared to proceed similarly for both types of reactors. This similarity was not preserved for Cu/Al_2_O_3_. The activation energies remained very low, confirming that the cyclization reaction was slow, which was also indicated by the Thiele modulus.

The changes of standard enthalpy of adsorption for both metals were negative for both metals, confirming an exothermic process. Vannice *et al.* [34] recommended a set of criteria to validate the thermodynamic properties extracted from the adsorption constant analysis. First, adsorption is invariably exothermic; thus, the enthalpy of adsorption is negative, *i.e.*, $∆H_{ads}^{0}$ < 0. Second, the entropy must decrease after adsorption, including the dissociative adsorption of a diatomic molecule; thus, $∆S_{ads}^{0}$ = $S_{ads}^{o}$ − $S_{l}^{o}$, where $S_{l}^{o}$ is the standard total entropy in the liquid phase. As a consequence of these rules, 0 < −$∆S_{ads}^{0}$ < $S_{l}^{o}$. The value of $S_{l}^{o}$ for phthalonitrile must be approximately 175 J/mol-K because the values for benzene and phthalic anhydride molecules are 173 and 179 J/mol-K, respectively. Based on the values obtained for both metals, both criteria were satisfied. Phthalonitrile was adsorbed with a greater energy on Ni/Al_2_O_3_ than on Cu/Al_2_O_3_ because of the standard enthalpy of adsorption, particularly that of NiO. Additionally, the mobility of the adsorbed molecule was similar for both surfaces because of the very similar values of the standard entropy of adsorption. This finding suggests that once the phthalonitrile molecule adsorbed onto the Ni–NiO/alumina or Cu/alumina surface, its mobility depended more on whether the functional groups included the same molecule. Furthermore, nitrile groups had to be available and the aromatic ring had to be adsorbed on the surface to begin to react with the nucleophilic agent (sodium methoxide).

### 2.7. FT-IR Spectra and Elemental Analysis of Metallic Phthalocyanine Products

**Figure 8:**
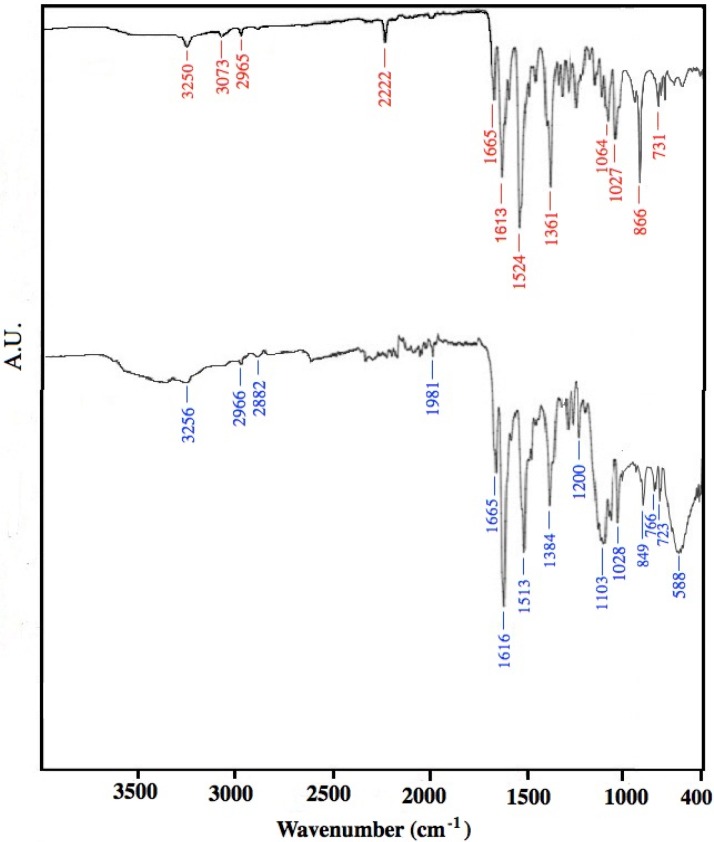
FT-IR spectra of (**a**) Ni-phthalocyanine; and (**b**) Cu-phthalocyanine obtained in parallel-plate reactor at 50 °C.

Figure 8 shows the FT-IR spectra for Ni- and Cu-phthalocyanine products (Figure 8a,b, respectively) obtained at 50 °C in the parallel-plate reactor. Table 3 presents the assignments of the FT-IR characteristic bands that correspond to a typical metal-phthalocyanine molecule [35,36].

Copper phthalocyanine exists in many polymorphic forms. The most commonly studied phases are alpha (α) and beta (β), and two less common crystalline forms are γ and ε. The resulting β-polymorph has a larger crystalline form, and large crystals change the shade and the tinctorial strength of paint during storage before its final use. Therefore, the β-form is preferred in industrial use.

The different crystalline forms can be identified in the finger region of FT-IR spectra [35,36,37]. The peaks at 731, 866, and 1027 cm^−1^ of the Ni-phthalocyanine spectra (Figure 8a) can be attributed to the α phase. The Cu-phthalocyanine spectra (Figure 8b) present these bands at approximately the same wavenumbers but also exhibit peaks at 766, 1103, and 118 cm^−1^, which have been reported for the β crystalline phase [37].The bands at ~3250 cm^−1^ were assigned to the N–H bond, which indicates the presence of free metal phthalocyanine; however, these bands were not significantly intense.

**Table 3:** Assignments of the FT-IR characteristic peaks of the NiPc and CuPC products.

Wavenumber cm^−1^		Assignment
NiPc	CuPc	
3250	3256	N–H stretching
3073	3050	*sp^2^* C–H stretching
2965, 2870	2966, 2882	*sp^3^* C–H stretching
2222	1981	C–H overtones
1655	1655	N–H bending
1613	1616	benzene ring stretching
1524	1513	benzene in-plane deformation
1361	1384	C–O deformation
1320*	1320*	C=N–C at bridge sites
1268*	1269*	C=N–C at bridge sites
1195*	1196*	C–O stretching, O–H in-plane deformation
1164*	–	Ni–N
–	1196*, 1133*	Cu–N
1118*	1118*	C–H in-plane deformation, benzene in-plane deformation
1064	–	C–H in-plane deformation
–	1103	C–H in-plane deformation
–	766	C–H non-planar vibrations (out-of-plane bending)
731	723	C–H nonplanar deformation

*= Not signaled in Figure 8.

The percentages of elemental analysis of Cu-phthalocyanine results were (analyzed/theoretical) Cu = 10.25/11.03 wt %, C = 65.07/66.72 wt %, N = 18.55/19.45 wt % and H = 2.05/2.80 wt %; for Ni-phthalocyanine were Ni = 9.55/10.28 wt %, C = 67.01/67.28 wt %, N = 18.90/19.62 wt %, and H = 2.41/2.82 wt %. According to elemental analysis results, compositions of the samples of the obtained products correspond to typical metal phthalocyanines of ~96%–98% purity.

## 3. Experimental Section

### 3.1. Preparation and Characterization of Supported Elemental Metals

The copper or nickel samples supported on alumina were synthesized as reported in our previous work [12]. The samples prepared in this work were characterized by different techniques. The crystal phases of all samples were characterized by X-ray diffraction (XRD) using a Siemens D-5000 instrument (Siemens Inc., Berlin, Germany) with Cu Kα radiation at a scan rate of 0.05° at 2θ min^−1^ and 40 kV/30 mA. The surface areas of the powders were measured using a Quantachrome Autosorb-1 surface area analyzer (Boynton Beach, FL, USA). Nitrogen adsorption isotherms were obtained at 77 K after the samples were degassed below 10^−3^ Torr and 200 °C for 8 h. SEM photomicrographs were obtained to study the M/Al_2_O_3_ (M = supported Ni or Cu particles) morphology. A Carls Zeiss DSM950 model was used at 30 kV/50 mA and with a 9 mm working distance.

### 3.2. Reaction Conditions in Batch and Parallel Reactors

Batch experiments: The tests were conducted as reported in our previous work [12]. For this work the reaction time was 24 h at 150 rpm and a constant temperature. The experiments were conducted at 30, 40, and 50 °C.

**Figure 9:**
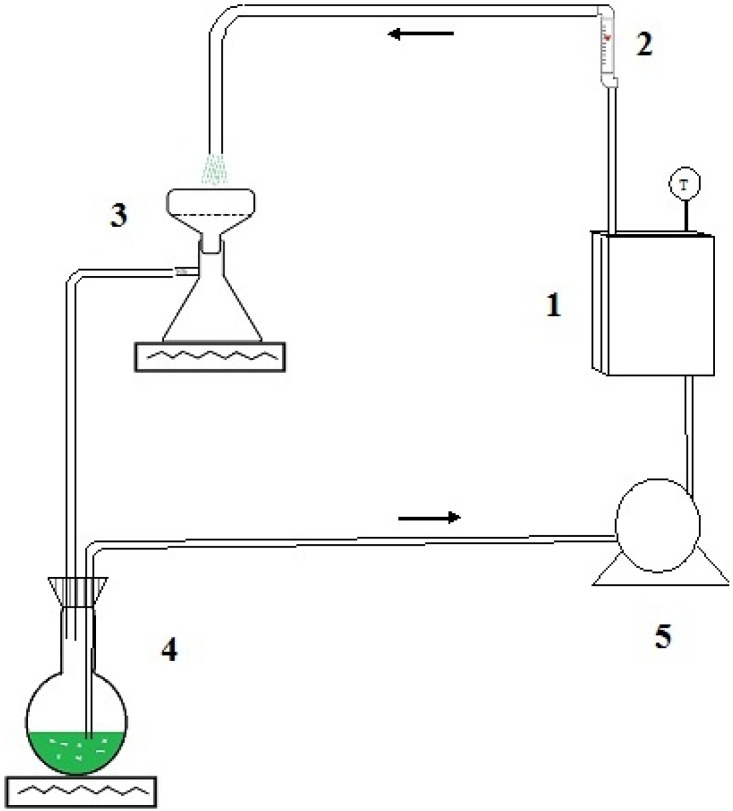
Experimental set up for parallel reactor tests. (**1**) Parallel-plate reactor; (**2**) rotameter; (**3**) filter flask with electric heat; (**4**) round bottom flask with stirring and electric heat; and (**5**) peristaltic pump.

Parallel-plate reactor experiments: Two removable inner plates were coated with a layer of commercial adhesive, Devcomplasteel^®^, as uniformly as possible. A weighed amount of Ni/Al_2_O_3_ or Cu/Al_2_O_3_ powder was immediately spread evenly over the entire surface of each plate and allowed to dry for 24 h. Once the adhesive dried, the excess M/Al_2_O_3_ powder was removed. The amount of remaining M/Al_2_O_3_ was calculated by the weight difference between the initially added and removed M/Al_2_O_3_. The weight of the M/Al_2_O_3_ adhered on the reactor plates, was used to calculate the amounts of precursor phthalonitrile and CH_3_ONa to maintain the same proportions used in the batch reactor. The experimental setup for the parallel-plate reactor tests is shown in Figure 9. The tests were conducted at 2000 mL/min in circulation for 24 h at constant temperature in 10 °C increments from 30 to 60 °C. The MPc solid product was retained on the filter paper of the flask while the solution recirculated through the parallel-plate reactor. After the elapsed reaction time, the MPc on the filter paper was removed, and the solution was filtered and washed several times with ethanol to recover traces of the product. The FT-IR spectra for MPc in KBr mixed disks were determined using a Perkin-Elmer Paragon 1000 PC spectrometer (PerkinElmer, Inc., Waltham, MA, USA). An organic microanalyzer Perkin-Elmer 2400 Series II CHNS/O system was used for C, H and N content determination. Ni and Cu elemental analysis was carried out using Flame Atomic Adsorption Spectometry.

## 4. Conclusions

Nickel and copper phthalocyanine were synthesized in ethanol at low temperatures (30–60 °C) from phthalonitrile and metallic copper and nickel nanoparticles, which were deposited on alumina with sodium methoxide as a nucleophilic agent. Two reactor configurations were used: a batch reactor (a flask) and a parallel-plate reactor, in which the Ni/ or Cu/alumina powders were fixed and the ethanolic solution was cycled. The Ni and Cu metals on alumina were characterized as nanoparticles (30–150 nm) in agglomerations of 1 to 1.5 μm, with Ni having a lower mean crystallite sizes than Cu (46 and 169 nm, respectively) in XRD patterns analysis. NiO particles were identified in the Ni/Al_2_O_3_ sample, which influenced the morphology. The Cu/Al_2_O_3_ sample only presented the pure metal phase and had a more homogenous morphology than the nickel sample. Both powder samples presented mesopores and some macropores, which could primarily be attributed to the alumina used as the support. However, the copper particles blocked the macropores and some of the larger mesopores in the Cu/Al_2_O_3_ sample, leading to a lower specific area than that of the Ni/Al_2_O_3_ sample. Both samples contained smaller mesopores that were sufficiently accessible for the percolation of reactants.

The activation energies for the second-order kinetic expression for both samples in the batch reaction were very similar, which primarily indicates that the cyclization reaction to form the metallic phthalocyanine from four phthalonitrile molecules with a metal was slow. The effect of mass transfer was evaluated in the parallel-plate reactor tests using a three-resistance model plotted as a function of the Thiele modulus. The Thiele modulus values were less than 10^−1^ for the studied temperatures in the parallel-plate reactor, which is representative of slow reactions and kinetic control. A Langmuir-Hinshelwood model was fitted to the experimental data in the parallel-plate reactor, and the kinetic parameters were analyzed. This model considered that the adsorption phthalonitrile to the catalytic surface occurred in one step and that the surface reaction was the controlling step. For both metal/alumina surfaces, the activation energies of the surface reactions were, again, very low, confirming that the cyclization reaction on the surface was slow. The thermodynamic properties for the reversible adsorption constant of the model included exothermic enthalpy values, which were more negative for the Ni/Al_2_O_3_ sample than for the Cu/Al_2_O_3_. The surface morphology and composition of the Ni/Al_2_O_3_ sample influenced this thermodynamic property, whereas the standard entropies of adsorption were very similar for both samples. The Ni/Al_2_O_3_ sample was more reactive than the Cu/Al_2_O_3_ sample, influenced for the NiO morphology and phthalonitrile adsorption. The crystalline form of the metallic phthalocyanine products contained more type α than β material for the low-temperature reaction conducted on the parallel-plate reactor.

## Nomenclature

*C_Ain_*: Concentration of *A* entering the channel (mol/dm^3^)
*C_Aout_*: Concentration of *A* exiting the channel (mol/dm^3^)
*C_As_*: Concentration of *A* on the washcoat surface (mol/dm^3^)
*C_Aw_*: Concentration of *A* in the washcoat bulk (mol/dm^3^)
*C_AB_*: Bulk concentration of *A* in the fluid phase (mol/dm^3^)
*Sh_e_*: External Sherwood number
*Sh_i_*: Internal Sherwood number
ϕ: Thiele modulus
*z*: Channel height (dm)
Ω_1_, Ω_2_: Cross-sectional domain of the fluid phase and washcoat
$R_{\Omega_{1}}$, $R_{\Omega_{2}}$: Effective transverse diffusion length for the fluid phase and washcoat (=*A*_Ω_/*P*_Ω_) (dm)
*C_s_*: Concentration at the surface (mol/dm^3^)
*R*(*C_s_*): Reaction rate by reactor volume (mol/dm^3^-s)
*D_e_*: Effective diffusivity (dm^2^/s)
*R*(*C*´): Reaction rate by catalyst volume (mol/dm^3^-s)
ε: Porosity of the catalyst in the tubular reactor
τ: Tortuos ity of the pores of the catalyst
*D_p_*: Diffusion coefficient in the pores (m^2^/s)
*D_K_*: Knudsen diffusion coefficient (m^2^/s)
*d_p_*: Average pore diameter (m)
*D_AB_*: Molecular diffusivity of specie A in solvent B (dm/s^2^)
φ: Association factor for the solvent, 1.5 for ethanol as a solvent.
*M*: Molar mass of the diffusing species (g/mol)
*T*: Temperature of the diffusing species (K)
*M_B_*: Molar mass of solvent (g/mol)
μ: Solution viscosity (g/dm-s)
ν*_A_*: Molar volume of the solute in the normal boiling point (dm^3^/mol)
*Sh_app_*: Experimentally observable Sherwood number
*L*: Length/distance along the monolith channel
*Sh(L)*: Sherwood number as function of length/distance along the monolith channel
*P*: Transverse Peclet number
*Sc*: Schmidt number (=ν/*D_B_*)
υ: Kinematic viscosity (dm^2^/s)
*D*_h_: Channel hydraulic diameter (=4$R_{\Omega_{1}}$)
*Sh*_*e*∞_: Asymptotic external Sherwood number
*Sh*_*i*∞_: Asymptotic internal Sherwood number
*Λ*: Constant for the calculation of the internal Sherwood number
$C_{A_{0}}$: Concentration of *A* at time zero (mol/dm^3^)
*x*: Fraction conversion at time t
g*_cat_*: Grams of catalyst
ν*_reactor_*: Volume of reactor (dm^3^)
*ρ_B_*: Bulk density of catalyst charged by reactor volume (g*_cat_*/ν*_reactor_*)
*A_o_*: Pre-exponential constant in second-order kinetics (dm^3^/mol^2^-s)
*E_a_*: Activation energy in second-order kinetics (J/mol)
*t*: Elapsed reaction time (s)
$-r_{A}^{´}$: Surface reaction rate (mol/s-g*_cat_*)
*F_Ao_*: Molar flow of A (mol/s)
*W_cat_*: Weight of catalyst (g)
*k*: Kinetic constant of surface reaction of Langmuir-Hinshelwood expression (dm^3^/s-g*_cat_*)
*K_A_*: Equilibrium constant for adsorption of Langmuir-Hinshelwood expression (dm^3^/mol)
*k*_0_: Pre-exponential constant of Arrhenius expression for k (dm^3^/s-g*_cat_*)
*E_aP-PR_*: Activation energy of Langmuir-Hinshelwood expression for parallel-plate reactor (J/mol)
$∆H_{\text{ads}}^{0}$: Change standard enthalpy of adsorption (J/mol)
$∆S_{\text{ads}}^{0}$: Change standard entropy of adsorption (J/mol-K)
$S_{\text{ads}}^{0}$: Standard total entropy of adsorption (J/mol-K)
$S_{l}^{0}$: Standard total entropy in the fluid phase (J/mol-K)
